# An ultrasound-based nomogram model in the assessment of pathological complete response of neoadjuvant chemotherapy in breast cancer

**DOI:** 10.3389/fonc.2024.1285511

**Published:** 2024-03-04

**Authors:** Jinhui Liu, Xiaoling Leng, Wen Liu, Yuexin Ma, Lin Qiu, Tuerhong Zumureti, Haijian Zhang, Yeerlan Mila

**Affiliations:** ^1^ Department of Ultrasound, The Tenth Affiliated Hospital of Southern Medical University (Dongguan People’s Hospital), Dongguan, Guangdong, China; ^2^ Artificial Intelligence and Smart Mine Engineering Technology Center, Xinjiang Institute of Engineering, Urumqi, China; ^3^ Department of Ultrasound, The Affiliated Tumor Hospital of Xinjiang Medical University, Urumqi, Xinjiang, China

**Keywords:** nomogram, radiomics, breast cancer, ultrasound, neoadjuvant chemotherapy

## Abstract

**Introduction:**

We aim to predict the pathological complete response (pCR) of neoadjuvant chemotherapy (NAC) in breast cancer patients by constructing a Nomogram based on radiomics models, clinicopathological features, and ultrasound features.

**Methods:**

Ultrasound images of 464 breast cancer patients undergoing NAC were retrospectively analyzed. The patients were further divided into the training cohort and the validation cohort. The radiomics signatures (RS) before NAC treatment (RS1), after 2 cycles of NAC (RS2), and the different signatures between RS2 and RS1 (Delta-RS/RS1) were obtained. LASSO regression and random forest analysis were used for feature screening and model development, respectively. The independent predictors of pCR were screened from clinicopathological features, ultrasound features, and radiomics models by using univariate and multivariate analysis. The Nomogram model was constructed based on the optimal radiomics model and clinicopathological and ultrasound features. The predictive performance was evaluated with the receiver operating characteristic (ROC) curve.

**Results:**

We found that RS2 had better predictive performance for pCR. In the validation cohort, the area under the ROC curve was 0.817 (95%CI: 0.734-0.900), which was higher than RS1 and Delta-RS/RS1. The Nomogram based on clinicopathological features, ultrasound features, and RS2 could accurately predict the pCR value, and had the area under the ROC curve of 0.897 (95%CI: 0.866-0.929) in the validation cohort. The decision curve analysis showed that the Nomogram model had certain clinical practical value.

**Discussion:**

The Nomogram based on radiomics signatures after two cycles of NAC, and clinicopathological and ultrasound features have good performance in predicting the NAC efficacy of breast cancer.

## 1 Introduction

Breast cancer is the leading cause of cancer worldwide in 2020, which has become the “world’s number one cancer”. Highly aggressive breast cancer is difficult to treat and has a high recurrence rate and poor prognosis (1, 2). At present, neoadjuvant chemotherapy (NAC) is the standard treatment regimen for breast cancer, which can effectively reduce tumor volume and clinical stage (3). The efficacy evaluation of NAC determines the individualized treatment plan. However, the efficacy evaluation of NAC is still difficult at present.

The current efficacy evaluation methods for NAC mainly include pathological evaluation and clinical evaluation. Pathological evaluation is the gold standard for evaluating the efficacy of NAC in breast cancer (4), but it has a lag, and cannot provide timely guidance for clinical treatment. Ultrasound, as one of the main clinical evaluation methods, is more frequently used in NAC assessment than MRI and mammography (5). However, ultrasound lacks quantitative parameters compared with other imaging examinations.

In recent years, radiomics has shown potential advantages in improving the precise diagnosis of breast cancers, assessment of lymph node metastasis, and prognosis prediction (6). Ultrasound imaging combined with radiomics can achieve a timely and accurate quantitative assessment of the efficacy of NAC in breast cancer (7). For the time point to evaluate the efficacy, one study has shown that the use of pre-NAC ultrasound images of breast cancer patients can more accurately predict the efficacy of NAC (8). However, according to the Breast Cancer Diagnosis and Treatment Guidelines by the Chinese Anti-Cancer Association, the efficacy evaluation by ultrasound after two cycles of NAC has significantly improved accuracy (9, 10). Another study reported that if the efficacy was assessed as non-pathological complete response (pCR) after two cycles of NAC, and then NAC was replaced with other treatment regimens, the long-term prognosis of patients was improved (11). However, there are few reports on the use of ultrasound images after two cycles of NAC to predict its efficacy. Breast cancer before NAC often presents all malignant signs on ultrasound, while the malignant signs of breast cancer after NAC often disappear completely on ultrasound, resulting in the fact that breast cancers with different prognoses and curative effects before and after NAC treatment often have the same ultrasound signs (12, 13), and making it difficult to distinguish different prognoses using ultrasound signs. Ultrasound radiomics can extract more ultrasound signs that are invisible to the naked eye and can provide more information than conventional ultrasound (14).

Herein, we predicted the pCR of NAC in breast cancer patients. The ultrasound radiomics of breast cancer before and after NAC were extracted and their value in predicting pCR was analyzed. Furthermore, a Nomogram was constructed based on clinicopathological features, ultrasound features, and radiomics models. Our findings may help clinicians to optimize the individualized treatment for NAC patients promptly.

## 2 Materials and methods

### 2.1 Patients

**Figure 1:**
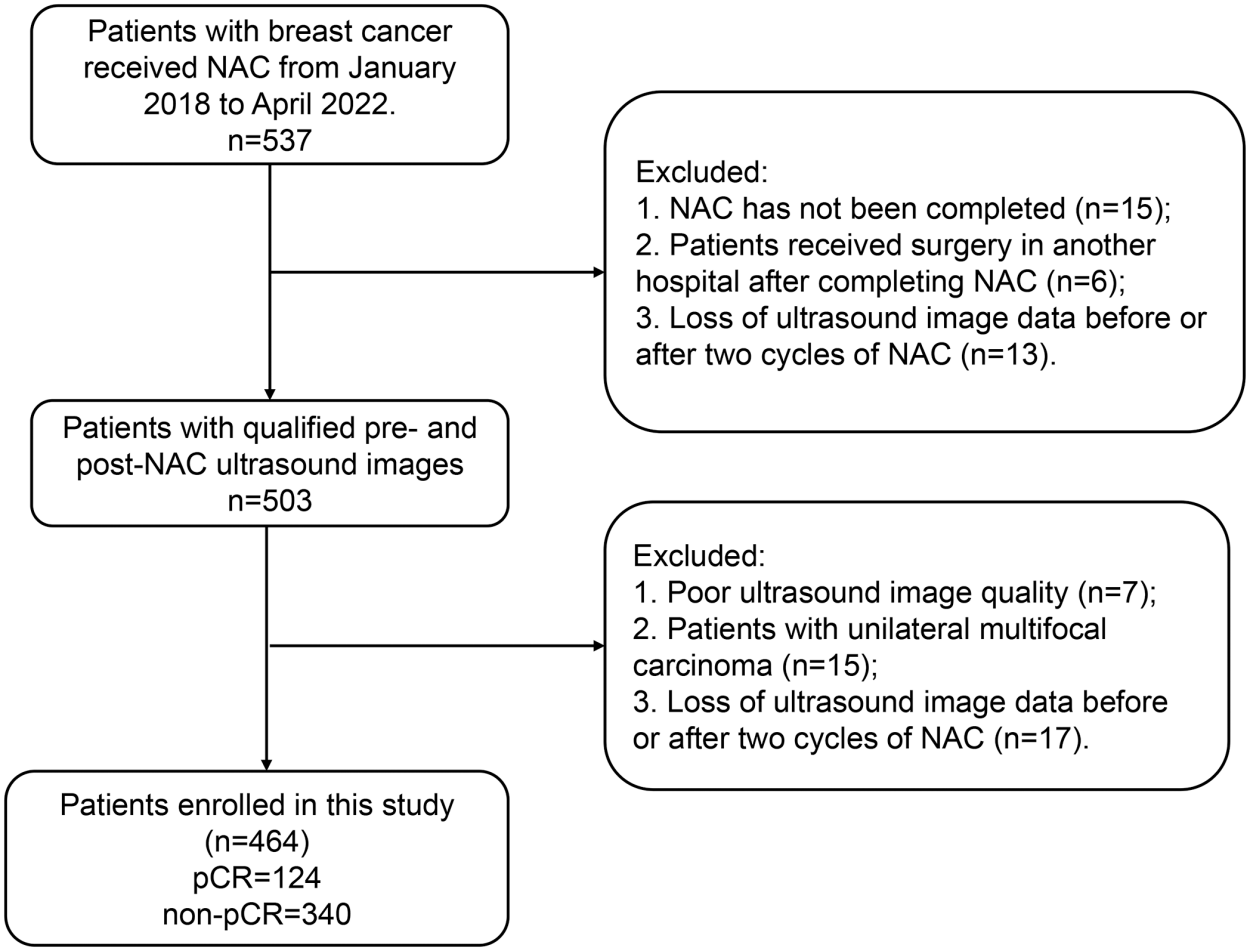
Flowchart of patient enrollment.

This study included patients who were diagnosed with breast cancer and who were admitted to the Tumor Hospital of Xinjiang Medical University between January 2018, and April 2022. The breast cancer diagnosis was confirmed by surgery and pathology. Inclusion criteria: (I) patients who had pathologically confirmed pCR or non-pCR after NAC; (II) patients who only received complete NAC therapy; (III) patients who underwent breast ultrasonography before surgery and after two cycles of NAC. Exclusion criteria: (I) patients with unavailable pathology results; (II) patients who did not complete NAC; (III) patients with insufficient ultrasound image quality; (IV) patients who had unilateral multifocal carcinoma. The flowchart of patient enrollment is shown in **Figure 1**. The study was conducted following the Declaration of Helsinki and approved by the ethics committee of Tumor Hospital of Xinjiang Medical University (approval number G-2023027). The written informed consent was obtained from each patient.

We randomly assigned the finally enrolled 464 patients with breast cancer into the training cohort (n=324) and the validation cohort (n=140). In the training cohort, 84 patients had pCR and 240 patients had non-pCR. In the validation cohort, 40 patients were with pCR and 100 patients were with non-pCR.

### 2.2 NAC and pathological evaluation of NAC efficacy

Treatment regimens and schedules followed the National Comprehensive Cancer Network (NCCN) guidelines. The NAC regimen was based on anthracyclines and taxanes (15).

All patients underwent standard histopathological examination to assess their response to NAC. The criteria for pCR were no residual invasive carcinoma in the specimen (residual ductal carcinoma *in situ* may be present) and no lymph node involvement in the ipsilateral sentinel lymph node or axillary lymph node.

### 2.3 Data collection

Clinical data collection included patient age, tumor types (e.g., invasive ductal carcinoma, invasive lobular carcinoma), presence of vascular invasion (positive or negative), TNM staging (stages I, II, and III), T staging [stages 1-4), N staging (stages 0-3), histological grade (low grade (I, II) and high grade (III)], estrogen receptor (ER) (positive or negative), progesterone receptor (PR) (positive or negative), human epidermal growth factor receptor 2 (Her2) (positive or negative), and Ki-67 expression (< 20% or ≥ 20%). TNM staging adhered to the 2017 AJCC Eighth Edition TNM Staging Standard for breast cancer. Ultrasound data collection encompassed post-NAC tumor characteristics, such as shape (regular or irregular), position (parallel or not parallel to the skin), margins (regular or irregular), internal echo (homogeneous or non-homogeneous), posterior echo (iso-echoic or weakened-echoic), calcifications (coarse, fine, or none), distortion of surrounding structures (distorted or not distorted), blood flow (internal type, peripheral type, or none), breast background (fatty echo or fibrous echo), as well as changes in the long and anterior-posterior diameters of tumor before and after treatment (< 30% or ≥ 30%).

### 2.4 Ultrasound examination

Ultrasound examination was performed with GE Logic E9 with the high-frequency linear array probe L-16-5. The ultrasound images with the longest diameter were selected for analysis. Two radiologists (with at least 10 years of experience in breast ultrasound), who were blinded to the pathological findings, delineated the region of interest (ROI) in the ultrasound images by using Itk-Snap (version 3.8.0). The interclass correlation coefficient (ICC) was used to assess the agreement of the feature extraction between observers and within observers. Ratings of ICC were assigned as follows: an ICC of less than 0.40 was considered ‘Poor’, 0.40–0.59 was labeled ‘Fair’, 0.60–0.74 was categorized as ‘Good’, and 0.74–1.00 was deemed ‘Excellent’.

### 2.5 Extraction of radiomics features

**Figure 2:**
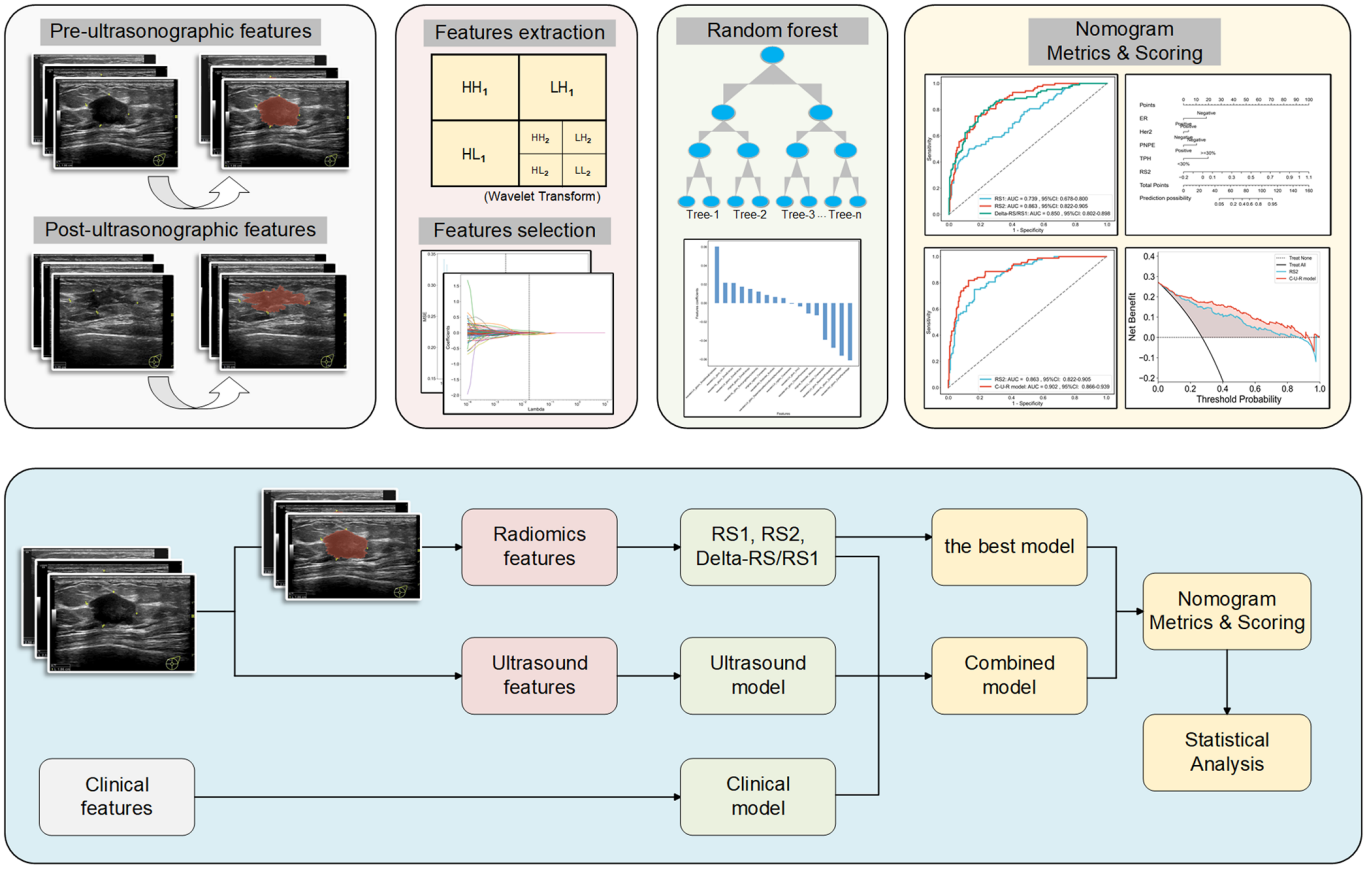
Flowchart of radiomics feature extraction and model establishment.

The flowchart of radiomics feature extraction and model establishment is shown in **Figure 2**. In detail, the ultrasound features were extracted from ultrasound images using the PyRadiomics open-source tool (https://pyradiomics.readthedocs.io/en/latest/index.html). The ultrasound images were processed using the Wavelet filter. A total of 7 categories of features were extracted, including 1) First Order Features; 2) Shape Features; 3) GLCM Features; 4) GLSZM Features; 5) GLRLM Features; 6) NGTDM Features; and, 7) GLDM Features. The radiomics signatures (RS) of ultrasound images before NAC (defined as RS1), and those after 2 cycles of NAC (defined as RS2) were obtained. The different signatures between RS1 and RS2 were defined as Delta-RS/RS1.

### 2.6 Establishment and performance evaluation of radiomics models

We used the PyRadiomics open-source tool (https://pyradiomics.readthedocs.io/en/latest/index.html) to establish radiomics models. Before the feature selection, the ICC was calculated to ensure the repeatability and stability of features with a threshold of 0.75. All signatures were normalized by the Z-score method. Student’s test and Pearson correlation analysis were performed. The Least Absolute Shrinkage and Selection Operator (LASSO) was used to further screen the signatures, and according to the Minimum Squared-Error criterion, the signatures with the greatest correlation were selected. The Random Forest classifier was used to analyze the key signatures for predicting pCR, and the 10-fold cross-validation was used to optimize hyperparameters, thus improving model performance.

According to the calculation formula in the official documentation of Radiomics, the Rad-score of each patient was calculated as follows:


Rad−score=β0+β1X1+β2X2+β3X3+…+βnXn.

Xn represents the RSs after screening, β0 is the constant of the Rad-score, and βn is the regression coefficient of the corresponding RS in the regression model (16).

In detail, the formula for the Rad-score of RS1 was 0.301724 + 0.018033×original_glrlm_GrayLevelNonUniformityNormalized-0.004469×original_ngtdm_Strength+0.004457×original_gldm_SmallDependenceHighGrayLevelEmphasis+0.027550×wavelet-LLH_firstorder_Mean+0.090245×wavelet-LHH_ngtdm_Coarseness+0.043016×wavelet-HLL_firstorder_MeanAbsoluteDeviation+0.045260×wavelet-HLH_glcm_DifferenceVariance-0.004468×wavelet-HLH_glszm_LargeAreaLowGrayLevelEmphasis+0.015469×wavelet-HLL_glszm_ZoneEntropy+0.002321×wavelet-HHL_ngtdm_Strength-0.005785×wavelet-LLH_glcm_ClusterShade-0.010428×wavelet-LLH_glcm_MaximumProbability.

The formula for the Rad-score of RS2 was 0.267241-0.010622×original_glcm_ClusterShade-0.022649×original_glszm_SmallAreaHighGrayLevelEmphasis+0.048624×original_ngtdm_Strength+0.010071×wavelet-LLH_firstorder_Mean+0.022298×wavelet-LLH_glcm_MaximumProbability+0.100730×wavelet-LHH_ngtdm_Coarseness-0.005865×wavelet-LHH_gldm_DependenceNonUniformityNormalized+0.005142×wavelet-HLL_firstorder_Mean+0.027327×wavelet-HHL_ngtdm_Strength+0.017414×wavelet-HHL_glszm_ZonePercentage+0.040358×wavelet-HHL_gldm_SmallDependenceEmphasis+0.020727×wavelet-LHL_firstorder_10Percentile-0.051783×wavelet-LHL_glcm_Idn-0.009552×wavelet-LLH_glrlm_LongRunLowGrayLevelEmphasis-0.001952×wavelet-LLH_glrlm_RunEntropy+0.057903×wavelet-LLL_ngtdm_Coarseness+0.001388×wavelet-LLL_ngtdm_Contrast.

The formula for the Rad-score of Delta-RS/RS1 was 0.301724 + 0.012994*original_firstorder_Minimum+0.015151*original_glszm_ZoneEntropy+0.008663*original_ngtdm_Complexity-0.003557*wavelet-LHL_glcm_Imc2-0.047644*wavelet-LHL_glcm_MaximumProbability+0.060348*wavelet-LHH_glszm_SmallAreaEmphasis+0.017589*wavelet-LHH_glszm_ZoneEntropy-0.000699*wavelet-LHH_ngtdm_Coarseness+0.005423*wavelet-LLH_gldm_DependenceNonUniformityNormalized+0.021508*wavelet-LHL_glszm_ZoneEntropy-0.056111*wavelet-HLL_glszm_ZonePercentage+0.012303*wavelet-HLL_glrlm_RunLengthNonUniformity+0.006602*wavelet-HHL_firstorder_Kurtosis-0.011061*wavelet-HHL_glcm_ClusterProminence-0.061069*wavelet-HHH_glszm_ZonePercentage+0.021748*wavelet-LLH_glcm_Idmn-0.039115*wavelet-LLL_ngtdm_Coarseness.

### 2.7 Construction and performance evaluation of radiomics nomogram

The Nomogram was constructed based on the optimal radiomics model, and the significant clinicopathological features and ultrasound features affecting pCR. The Nomogram and the radiomics model were compared with the DeLong test. Decision curve analysis (DCA) was used to calculate and compare the net benefit at different threshold probabilities for the training and validation cohorts to assess the clinical value of the radiomics model and Nomogram.

### 2.8 Statistical analysis

Statistical analysis was performed using Python (version 3.7) and R language (version 4.2.0). The data of normal distribution and non-normal distribution were analyzed by t-test and Mann-Whitney U test, respectively. Enumeration data were analyzed by chi-square test. The significant clinicopathological features and ultrasound features affecting pCR were screened with univariate and multivariate analysis. The performance of each model was assessed using receiver operating characteristic (ROC) curves. The area under the ROC curve (AUC) was calculated. A two-tailed p-value <0.05 indicated statistical significance.

## 3 Results

### 3.1 Clinicopathological and ultrasound features of patients

A total of 464 patients were enrolled in this study. The clinicopathological features and ultrasound imaging features of the patients are shown in **Table 1**; **Supplementary Table S1**. In both training and validation cohorts, the ER status, Her2 status, vascular invasion, PR status, post-NAC posterior echo, percentage of ultrasound length, delta height, and percentage of ultrasound height were significantly associated with pCR (p<0.05). There was no significant association between pCR and other features.

**Table 1:** Baseline characteristics of the patients.

Characteristics	Training cohort			Validation cohort			P-value
	non-pCR (N=240)	pCR (N=84)	P-value	non-pCR (N=100)	pCR(N=40)	P-value	
**Age,** Mean (SD), years	48.8 (9.96)	46.9 (8.63)	0.306	47.2 (9.96)	48.8 (7.82)	0.662	0.768
**NAC duration,** Mean (SD), day	154 (78.9)	153 (55.5)	0.992	157 (88.8)	147 (24.1)	0.797	1
**Tumor type**			0.426			0.828	0.999
Invasive ductal carcinomas	214 (89.2%)	70.0 (83.3%)		87.0 (87.0%)	37.0 (92.5%)		
Invasive lobular carcinoma	6.00 (2.5%)	1.00 (1.2%)		3.00 (3.0%)	0 (0%)		
Others	20.0 (8.3%)	13.0 (15.5%)		10.0 (10.0%)	3.00 (7.5%)		
**Vascular invasion**			0.0034			0.0347	0.680
Positive	62.0 (25.8%)	7.00 (8.3%)		31.0 (31.0%)	4.00 (10.0%)		
Negative	178 (74.2%)	77.0 (91.7%)		69.0 (69.0%)	36.0 (90.0%)		
**Nerve invasion**			0.329			0.739	0.865
Positive	28.0 (11.7%)	5 (6.0%)		12.0 (12.0%)	0 (0%)		
Negative	212 (88.3%)	79.0 (94%)		88.0 (88.0%)	40.0 (100%)		
**TNM stage**			0.966			0.388	0.095
I	22.0 (9.2%)	10.0 (11.9%)		10.0 (10.0%)	1.00 (2.5%)		
II	88.0 (36.7%)	31.0 (36.9%)		46.0 (46.0%)	25.0 (62.5%)		
III	130 (54.2%)	43.0 (51.2%)		44.0 (44.0%)	14.0 (35.0%)		
**T stage**			0.552			0.478	0.912
1	53.0 (22.1%)	12.0 (14.3%)		27.0 (27.0%)	4.00 (10.0%)		
2	106 (44.2%)	48.0 (57.1%)		47.0 (47.0%)	26.0 (65.0%)		
3	45.0 (18.8%)	15.0 (17.9%)		15.0 (15.0%)	6.00 (15.0%)		
4	36.0 (15.0%)	9.00 (10.7%)		11.0 (11.0%)	4.00 (10.0%)		
**N stage**			0.0981			0.458	0.423
0	30.0 (12.5%)	23.0 (27.4%)		9.00 (9.0%)	7.00 (17.5%)		
1	117 (48.8%)	31.0 (36.9%)		57.0 (57.0%)	24.0 (60.0%)		
2	48.0 (20.0%)	14.0 (16.7%)		19.0 (19.0%)	2.00 (5.0%)		
3	45.0 (18.8%)	16.0 (19.0%)		15.0 (15.0%)	7.00 (17.5%)		
**Histological grading**			0.0023			0.719	0.298
Low grade invasive breast cancer(Grade I, II)	129 (53.8%)	51.0 (60.7%)		40.0 (40.0%)	19.0 (47.5%)		
High grade invasive breast cancer(Grade III)	111 (46.3%)	33.0 (39.3%)		60.0 (60.0%)	21.0 (52.5%)		
**ER status**			<0.001			<0.001	0.554
Positive	179 (74.6%)	37.0 (44.0%)		72.0 (72.0%)	14.0 (35.0%)		
Negative	61.0 (25.4%)	47.0 (56.0%)		28.0 (28.0%)	26.0 (65.0%)		
**PR status**			<0.001			0.0416	0.993
Positive	149 (62.1%)	25.0 (29.8%)		61.0 (61.0%)	15.0 37.5%)		
Negative	91.0 (37.9%)	59.0 (70.2%)		39.0 (39.0%)	25.0 (62.5%)		
**Her2 status**			<0.001			<0.001	0.662
Positive	49.0 (20.4%)	55.0 (65.5%)		20.0 (20.0%)	31.0 (77.5%)		
Negative	191 (79.6%)	29.0 (34.5%)		80.0 (80.0%)	9.00 (22.5%)		
**Ki-67 status**			0.067			0.824	0.584
< 20%	30.0 (12.5%)	3.00 (3.6%)		8.00 (8.0%)	2.00 (5.0%)		
≥ 20%	210 (87.5%)	81.0 (96.4%)		92.0 (92.0%)	38.0 (95.0%)		
**Rad-score for RS1,** Mean (SD)	0.229 (0.113)	0.365 (0.168)	<0.001	0.228 (0.113)	0.394 (0.216)	<0.001	0.941
**Rad-score for RS2,** Mean (SD)	0.188 (0.170)	0.490 (0.235)	<0.001	0.192 (0.183)	0.470 (0.247)	<0.001	0.967
**Rad-score for Delta-RS/RS1,** Mean (SD)	0.205 (0.137)	0.465 (0.224)	<0.001	0.196 (0.138)	0.397 (0.206)	<0.001	0.36

SD, standard deviation; ER, estrogen receptor; PR, progesterone receptor; Her2, human epidermal growth factor receptor 2; NAC, neoadjuvant chemotherapy; pCR, pathological complete response; RS, radiomics signature.

The chi-square test or Fisher’s exact test was used for the nominal variable, and the Mann–Whitney test was used for the continuous variable with the abnormal distribution. A two-tailed p-value <0.05 indicated statistical significance.

The significant clinicopathological and ultrasound features were subjected to multivariate logistic regression analysis (**Supplementary Tables S2**, **S3**). The results showed that ER status, PR status, Her2 status, post-NAC posterior echo, and percentage of ultrasound height were all significantly associated with higher pCR (p < 0.05). Patients with negative ER and Her2 or with Post-NAC posterior echo of Weaken-Echoic and percentage of ultrasound height ≥ 30% were easier to achieve pCR.

### 3.2 Screening and modeling of radiomics features

Through radiomics feature extraction, 851 radiomics features were screened from RS1, RS2, and Delta-RS/RS1, including 216 GLCM Features, 126 GLDM Features, 144 GLRLM Features, 144 GLSZM Features, 45 NGTDM Features, 162 First Order Features, and 14 Shape Features. Before selection, the ICC for the 369 features was >0.75, ensuring the repeatability of features. After screening by LASSO regression analysis, the results showed that when λ was 0.008685 (**Supplementary Figures S1A, B**), 0.003393 (**Supplementary Figures S1C, D**), and 0.017575 (**Supplementary Figures S1E, F**), the optimal models of RS1, RS2, and Delta-RS/RS1 could be obtained (**Supplementary Figure S1**).

A total of 12, 17, and 17 radiomics features with non-zero coefficients from RS1, RS2, and Delta-RS/RS1 were obtained, respectively (**Supplementary Figure S2**). Among the features with positive correlation coefficients, the optimal features of Coarseness (0.090245) and Difference Variance (0.045261) in RS1, Coarseness (0.057903) in RS2, and Small Area Emphasis (0.060348) and Idmn (0.021748) in Delta-RS/RS1 had the highest weight. Among the features with negative correlation coefficients, the optimal features of RS1 had a lower weight. IDN (inverse difference normalized) (-0.051783) in RS2 had the highest weight. Additionally, among the optimal features of Delta-RS/RS1, the features with the highest weight were Zone Percentage [-0.060348 (wavelet-HH), -0.056111 (wavelet-HL)], and Maximum Probability (-0.047644).

For the comparison of Rad-Score, it was shown that there was no significant difference in the Rad-score between the training cohort and the validation cohort (p>0.05, **Table 1**). Further univariate analysis showed that the pCR in breast cancer patients was closely related to the Rad-score (p<0.001, **Table 1**).

### 3.3 Prediction of NAC efficacy by radiomics, clinicopathological, and ultrasound models

**Figure 3:**
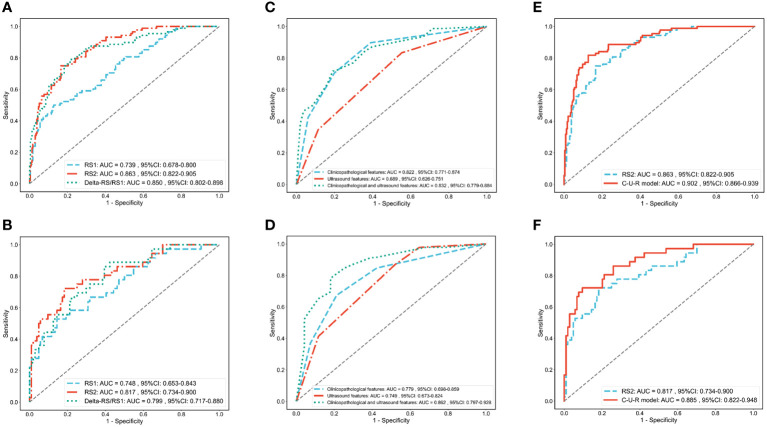
ROC analysis of each radiomics model, clinical and ultrasound model, and C-U-R model. ROC analysis of RS1, RS2, and Delta-RS/RS1 radiomics models in the training **(A)** and validation **(B)** cohorts. ROC analysis of clinicopathological features (clinical model), ultrasound features (ultrasound model), and the combination of the two (C-U model) in the training **(C)** and validation **(D)** cohorts. ROC analysis of the C-U-R model and RS2 model in the training **(E)** and validation **(F)** cohorts. C-U-R model, combined model of clinicopathological features, ultrasound features, and RS2; RS, radiomic signature; AUC, area under the ROC curve.

ROC was used to assess the role of the radiomics models in predicting the pCR status of breast cancer patients after two cycles of NAC. The Delong test showed that the performance of the RS2 (AUC_RS2_ = 0.863) was higher than the RS1 (AUC_RS1_ = 0.739, p _RS2 vs RS1_ = 0.002) but was no higher than Delta-RS/RS1 (AUC_Delta-RS/RS1_ = 0.850, p _RS2 vs Delta-RS/RS1_ = 0.682) in the training cohort (**Figure 3A**; **Table 2**). In the validation cohort, the performance of the RS2 (AUC_RS2_ = 0.817) was higher than the RS1 (AUC_RS1_ = 0.799, p _RS2 vs RS1_ = 0.213) but was no higher than Delta-RS/RS1 (AUC_Delta-RS/RS1_ = 0.748, p _RS2 vs Delta-RS/RS1_ = 0.689) (**Figure 3B**; **Table 2**).

**Table 2:** Performance comparison of RS1, RS2, Delta-RS/RS1, C-U-R model radiomics models.

	Training cohort				Validation cohort			
	RS1	RS2	Delta-RS/RS1	C-U-R model	RS1	RS2	Delta-RS/RS1	C-U-R model
AUC	0.739	0.863	0.850	0.902	0.748	0.817	0.799	0.885
Accuracy	0.787	0.830	0.806	0.861	0.793	0.829	0.779	0.857
Precision	0.721	0.762	0.712	0.795	0.667	0.731	0.620	0.767
Sensitivity	0.500	0.750	0.795	0.818	0.528	0.722	0.861	0.722
Specificity	0.873	0.835	0.780	0.873	0.856	0.817	0.606	0.904
Recall	0.352	0.546	0.477	0.659	0.389	0.528	0.361	0.639
F1-score	0.473	0.636	0.571	0.721	0.491	0.613	0.456	0.697
Youden Index	0.373	0.585	0.575	0.691	0.384	0.540	0.467	0.626

AUC, the area under the ROC curve; RS, radiomics signature; C-U-R, clinicopathological features, ultrasound features, and RS2.

The ROC curve evaluated the performance of clinicopathological and ultrasound features in predicting pCR (**Figures 3C, D**). In the training cohort, the AUC for clinicopathological and ultrasound features was 0.832 (95%CI: 0.779-0.884). In the validation cohort, the AUC for clinicopathological and ultrasound features was 0.862 (95%CI: 0.797-0.928).

We further evaluated the combined performance of clinicopathological features, ultrasound features, and RS2 (C-U-R model) and compared it with that of RS2 alone (**Table 2**). The results showed that in the training cohort, the C-U-R model (AUC_C-U-R model_ = 0.902) had a better performance than the RS2 (AUC_RS2_ = 0.863, p _C-U-R model vs RS2_ = 0.005) for predicting pCR (**Figure 3E**). In the validation cohort, the C-U-R model (AUC_C-U-R model_ = 0.885) also had a better performance than the RS2 (AUC_RS2_ = 0.817, p _C-U-R model vs RS2_ = 0.009) for predicting pCR (**Figure 3F**).

### 3.4 Construction and validation of the nomogram

**Figure 4:**
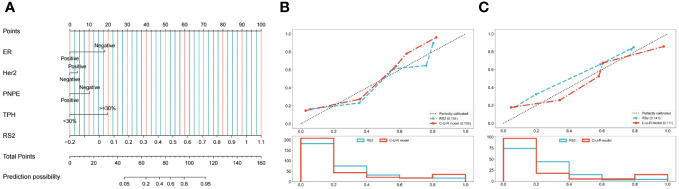
Development and performance of the nomogram. **(A)** Radiomics Nomogram was developed with vascular invasion, axillary lymph node metastasis, posterior echo delta-height/pre-height, and RS2 for the prediction of the probability of pCR. The predictors are ER, Her2, PNPE, TPH, and RS2. A vertical line was drawn from each predictor to ‘Points’ to get the score of the predictor. Then, the scores of each predictor were summed up. The ‘Total Points’ corresponded to the probability of pCR. Calibration curves of the model in the training **(B)** and validation **(C)** cohorts. The X-axis represents the predictive probability; the Y-axis denotes the observed probability. The 45° “Ideal” line represents the perfect prediction of the probability of pCR, and the “Bias-corrected” line indicates the prediction model of the nomogram. The closer the “Bias-corrected” line fits to the “Ideal” line, the better the discrimination of the nomogram is. ER, ER status; Her2, Human epidermal growth factor receptor 2; PNPE, Post-NAC posterior echo; TPH, Percentage of ultrasound length; RS, radiomic signature.

We constructed a Nomogram based on the C-U-R model. As shown in **Figure 4A**, the item “Points” represented the corresponding score of each variable. The calculated C-statistics of the Nomogram was 0.897, indicating the model had high predictive power. In addition, we used the Hosmer-Lemesow test to verify the calibration curves of the training cohort (**Figure 4B**) and the validation cohort (**Figure 4C**), and the results showed that the difference between the training cohort (p=0.50) and the validation cohort (p=0.97) was not statistically significant. We further used the DeLong test to compare the predictive power of the Nomogram and the RS2 radiomics model, which showed that the difference between the training cohort (p=0.005) and the validation cohort (p=0.009) was statistically significant. Additionally, based on the Youden index, the optimal critical score for the Nomogram was calculated as 71.742.

### 3.5 Clinical application of the nomogram model

We further used the DCA to compare the Nomogram model with the RS2 radiomics model (**Supplementary Figure S3**). With a threshold probability greater than 0.3%, the Nomogram model or RS2 benefited more than the “all-treated” or the “no-treatment” regimen. When the threshold probability was greater than 26.2%, the predictive ability of the Nomogram model was better than that of the RS2 radiomics model.

## 4 Discussion

In this study, the performance of clinicopathological features, ultrasound features, radiomics models, and Nomogram models in predicting pCR was analyzed and compared. The results showed that the Nomogram model was superior in predicting both the energy efficiency and clinical net benefit of pCR in patients.

### 4.1 Predictive performance of clinicopathological and ultrasound features

We found that the clinicopathological features had better predictive value of pCR than ultrasound features. This study showed that breast cancer with posterior echo attenuation had a lower pCR rate. It is known that the pathological basis of posterior echo attenuation is that the internal tumor stroma is rich and densely arranged (17). Therefore, non-triple negative breast cancer, which has rich tumor stroma, may have lower pCR rates (18). NAC can induce necrosis and fibrosis of breast cancer cells, leading to structure collapses (19). The change of the anteroposterior diameter of the lesion is much greater than the long diameter. Therefore, the larger change rate of the tumor anteroposterior diameter after treatment also indicates that the tumor necrosis rate is high, and it is easier to achieve pCR. However, the value of conventional ultrasound in predicting pCR by macroscopic signs is limited. Then, we tried to predict pCR by combining RSs.

### 4.2 Predictive performance of ultrasound radiomics models

Ultrasound imaging presents several notable advantages compared to other imaging modalities, including its wide availability, cost-effectiveness, real-time nature, non-invasiveness, and superior soft tissue resolution, which facilitates the accurate capture of fine structural details (20). Li et al. (21) utilized radiomics extracted from FDG PET/CT imaging to predict pCR in 100 cases of breast cancer patients who underwent NAC. Their retrospective analysis revealed that the combined model of clinical features and PET/CT imaging radiomics achieved an AUC of 0.958 in the training set and 0.730 in the validation set, surpassing the predictive accuracy of the clinical model. In addition, Liu et al. (22) found through a multicenter study involving 586 cases of breast cancer that the combined model of clinical features and multiparametric MRI radiomics predicted the pCR of breast cancer patients after NAC with significantly higher AUC compared to the clinical model. These studies confirm that the combined model based on imaging radiomics has high accuracy in predicting the efficacy of NAC in breast cancer, indicating the significant value of imaging radiomics in predicting the efficacy of breast cancer NAC. This study, analyzing retrospectively 464 breast cancer patients undergoing NAC, confirmed that the combined model based on ultrasound imaging radiomics also had high predictive efficacy for predicting the efficacy of breast cancer NAC, markedly outperforming the clinical model and yielding greater net benefits. Furthermore, we screened the RS1 and RS2, respectively, and found that the feature with the highest weight was Coarseness (0.090245/0.100730). Coarseness reflects the grayscale difference between a central pixel or voxel and its neighbors, thereby capturing the spatial rate of grayscale intensity changes (23). The results of this study showed that for both RS1 and RS2, the ultrasound images of patients with pCR had a lower rate of spatial change and more uniform local texture, and this change was more obvious for RS2. In addition, the weight of IDN in RS2 was -0.051783, whereas it was not present in RS1. IDN is another measure of the local homogeneity of an image. Unlike homogeneity, IDN normalizes the difference between the neighboring intensity values by dividing over the total number of discrete intensity values (24). These results suggest that patients with pCR and non-pCR have a larger difference in the local homogeneity of post-RS2 ultrasound images, which are not present in RS1. Meanwhile, the weight coefficient of the signature Difference Variance in RS1 was 0.045261, while it did not have any weight in RS2, suggesting that the effect of Difference Variance was diminished by NAC. The main reason for the above differences is the high tumor heterogeneity and disordered tumor cell arrangement before NAC treatment in breast cancer (25, 26), which is reflected in the RSs of pixel grayscale and texture inhomogeneity (27). After NAC treatment, patients with pCR will have a higher necrosis rate of tumor cells, lower tumor heterogeneity, and a uniform internal tissue structure, while patients with non-pCR will have less tumor cell necrosis and high tumor heterogeneity, which is not different than before treatment (28, 29). Consistently, we also found that the homogenization of texture feature was higher on ultrasound radiomics in patients with pCR response than in patients with non-pCR.

In this study, we used RS1 and RS2 radiomics to construct a radiomics model, and the best features selected included GLCM, NGTDM, GLSZM, GLRM, GLDM, and First Order features. Among them, GLCM and NGTDM features had the highest proportion. Studies have shown that GLCM reflects the changes in images and tumor heterogeneity by calculating the relative distance between the image and a specific pixel and by calculating the correlation coefficient of gray values in different directions (30–33). However, there are few studies on NGTDM. In this study, six types of texture features including GLCM, NGTDM, GLSZM, GLRM, GLDM, and First Order features were enrolled, and it was found that NGTDM and GLCM had similar weights. NGTDM quantifies the difference between a gray value and the average gray value of its neighbors within distance δ (30–34). Here, when we used NGTDM to evaluate the tumor ROI region, we also verified that NGTDM could accurately reflect tumor heterogeneity, thereby accurately predicting the NAC efficacy in patients.

In addition, we introduced a new radiomics model, Delta-RS/RS1, representing the magnitude of changes in ultrasound radiomics characteristics in breast cancer patients before and after NAC treatment. We found that among the positive correlation coefficients, the RSs with the highest weights were Small Area Emphasis (0.060348), Idmn (0.021748), and Zone Entropy (0.021508). Among the negative correlation coefficients, the RS with the highest weights was Zone Percentage (-0.060348 (wavelet-HH), -0.056111 (wavelet-HL)), followed by Maximum Probability (-0.047644). There were significant differences in these RSs between patients with pCR and non-pCR. In the positive correlation coefficient, the magnitude of change in patients with pCR was higher than that in patients without pCR. However, in the negative correlation coefficient, the magnitude of change in patients with pCR was lower than that in patients without pCR. The RS1, RS2, and Delta-RS/RS1 models all showed high predictive value for NAC response. However, the predictive value of RS2 and Delta-RS/RS1 was better than that of RS1. These results indicate that the radiomics model of breast cancer after two cycles of NAC can better predict the efficacy of NAC than that of before NAC. Clinicians should pay more attention to the radiomics characteristics of breast cancer after two cycles of NAC to facilitate the prediction of NAC efficacy.

### 4.3 Predictive performance of the C-U-R model and nomogram model

We further assessed the combined performance of the C-U-R model. We found that the C-U-R model had higher predictive performance for pCR than any single model. Then, we constructed a Nomogram model based on clinicopathological features, ultrasound features, and RS2. The Nomogram model showed accurate predictive power (C-statistics=0.897) in predicting NAC response. According to the Nomogram, after excluding RS2, the features with the highest individual scores were the percentage of ultrasound height ≥ 30% and negative ER status. We found that the optimal critical score for the Nomogram was 71.742. The breast cancer patients with a total score of > 71.742 were more likely to achieve pCR after NAC. In recent years, the Nomogram prediction model has been widely used in the clinic (33, 34). However, the indicators included in the Nomogram model for predicting the efficacy of NAC in breast cancer are confusing, and there is no conclusion on the evaluation time point of NAC. The indicators used for modeling in this study were more comprehensive, and the evaluation time point of NAC was determined to be after 2 cycles of NAC. Our results suggest that clinicians can comprehensively evaluate the efficacy of NAC according to the patients’ Nomogram score, ER status, Her2 status, etc., thus making the treatment strategy with the highest benefit to the patients.

### 4.4 Limitations

First, due to the individual differences of patients and to obtain high-quality images, the parameters of each ultrasound instrument during the examination were not unified. Therefore, different parameters of ultrasound may affect the final performance of the model. Secondly, the Delta-RS/RS1 was relatively new, and validation on Delta-RS/RS1 is needed. Finally, this study is a single-center retrospective study. Further multicenter studies are needed to assess the reliability of the Nomogram model.

### 4.5 Conclusions

In summary, the Nomogram model was developed based on clinicopathological features, ultrasound features, and RS2. The Nomogram model had good prediction performance of pCR after two cycles of NAC in breast cancer patients. Therefore, conventional clinicopathological features, and breast ultrasound features before NAC treatment and in the early stage of treatment (after two cycles of NAC) combined with radiomics can provide valuable prognostic information for predicting the efficacy of NAC in breast cancer and provide reference for making treatment strategies.

## Data availability statement

The raw data supporting the conclusions of this article will be made available by the authors, without undue reservation.

## Ethics statement

The studies involving humans were approved by the ethics committee of Tumor Hospital of Xinjiang Medical University. The studies were conducted in accordance with the local legislation and institutional requirements. The participants provided their written informed consent to participate in this study.

## Author contributions

JL: Conceptualization, Data curation, Formal analysis, Methodology, Software, Validation, Writing – original draft. XL: Conceptualization, Funding acquisition, Project administration, Supervision, Writing – review & editing. WL: Formal analysis, Software, Writing – review & editing. YXM: Investigation, Methodology, Writing – review & editing. LQ: Investigation, Methodology, Writing – review & editing. TZ: Investigation, Methodology, Writing – review & editing. HZ: Software, Writing – review & editing. YLM: Software, Writing – review & editing.
