# Supplementary material for: An ultrasound-based nomogram model in the assessment of pathological complete response of neoadjuvant chemotherapy in breast cancer
Source: Front Oncol. 2024 Mar 4;14:1285511. doi: 10.3389/fonc.2024.1285511 (PMC10946249; doi:10.3389/fonc.2024.1285511)
Supplement: Supplementary file 1 [file Image_1.pdf]

# Supplementary Figures

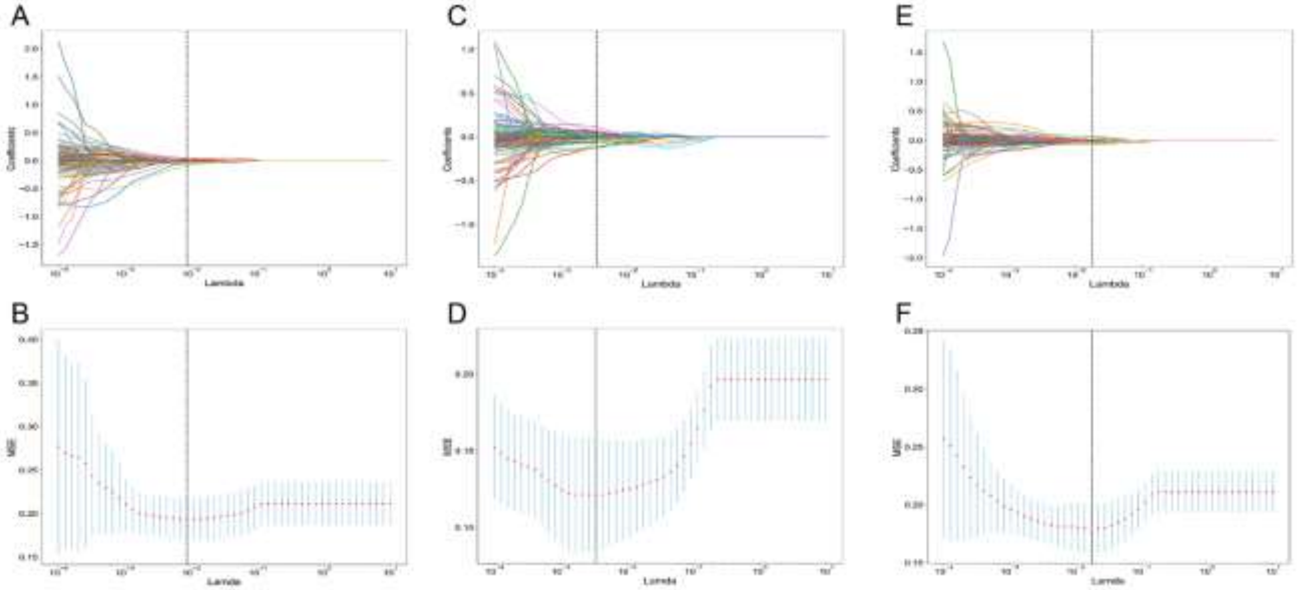

**Supplementary Figure S1. Determination of the optimal models of RS1, RS2, and Delta-RS/RS1.** (A) In RS1, the optimal value of 0.008685 was obtained. (B) LASSO coefficient profiles of the 12 radiomics features. A vertical line was drawn at the selected value using 10-fold cross-validation, where the optimal result was 12 non-zero coefficients. (C) In RS2, the optimal value of 0.003393 was obtained. (D) LASSO coefficient profiles of the 17 radiomics features. A vertical line was drawn at the selected value using 10-fold cross-validation, where the optimal result was 17 non-zero coefficients. (E) In Delta-RS/RS1, the optimal value of 0.017575 was obtained. (F) LASSO coefficient profiles of the 17 radiomics features. A vertical line was drawn at the selected value using 10-fold cross-validation, where the optimal result was 17 non-zero coefficients.

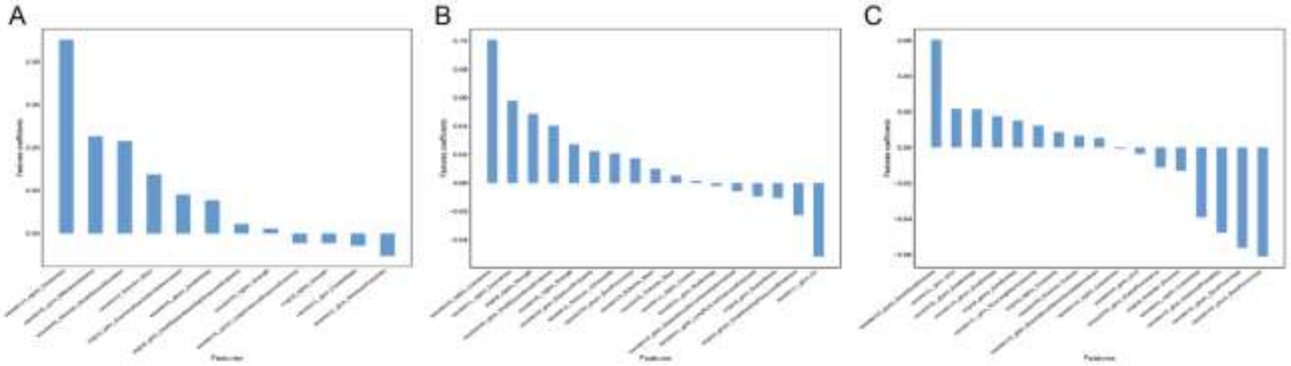


**Supplementary Figure S2. Results of feature selection in RS1, RS2 and Delta-RS/RS1.** The 12 nonzero coefficients features of RS1 (A), RS2 (B), and Delta-RS/RS1 (C) are presented.

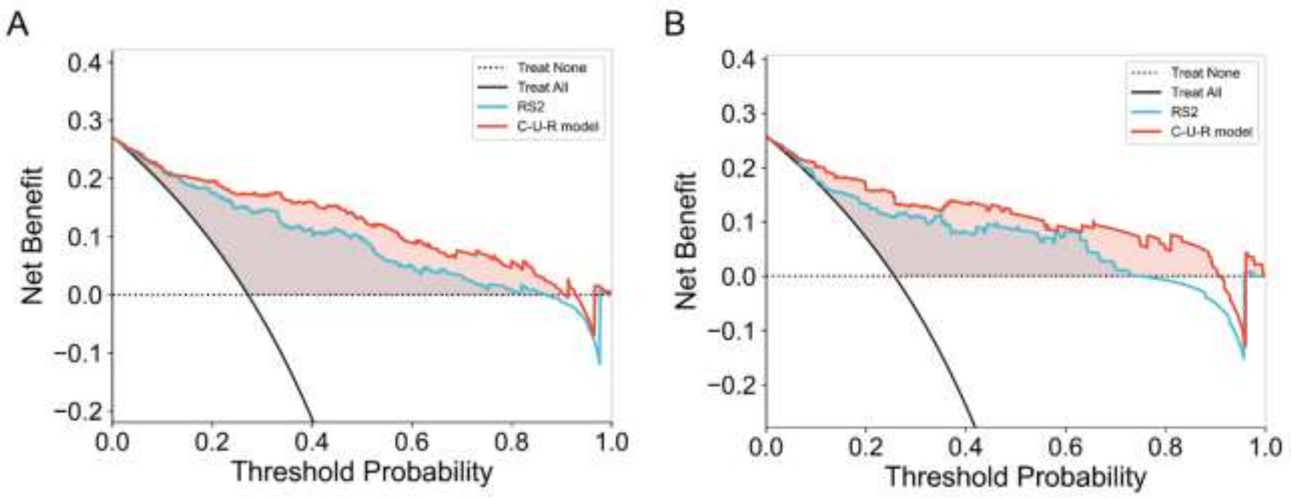


**Supplementary Figure S3. DCA for the Nomogram model and RS2 in training (A) and validation (B) cohorts.** The net benefit versus the threshold probability is plotted. The gray line represents the assumption that all patients reach pCR status; and the black line refers to the assumption that all patients reach non-pCR status. A model is only clinically useful if it has a higher net benefit than the default treat-all (all cases achieving pCR status) and treat-none (none of the cases achieving non-pCR) strategies.
