# Supplementary material for: An ultrasound-based nomogram model in the assessment of pathological complete response of neoadjuvant chemotherapy in breast cancer
Source: Front Oncol. 2024 Mar 4;14:1285511. doi: 10.3389/fonc.2024.1285511 (PMC10946249; doi:10.3389/fonc.2024.1285511)
Supplement: Supplementary file 2 [file DataSheet_1.pdf]

Supplementary Tables

Supplementary Table S1. Ultrasound characteristics of the patients.

	Training cohort			Validation cohort			P-value
	non-pCR (N=240)	pCR (N=84)	P-value	non-pCR (N=100)	pCR (N=40)	P-value	
**Post-NAC shape**			0.865			0.488	0.865
Irregular	212 (88.3%)	76.0 (90.5%)		85.0 (85.0%)	37.0 (92.5%)		
Regular	28.0 (11.7%)	8.00 (9.5%)		15.0 (15.0%)	3.00 (7.5%)		
**Post-NAC position**			0.0575			0.796	0.778
Non-Parallel	3.00 (1.3%)	5.00 (6.0%)		1.00 (1.0%)	1.00 (2.5%)		
Parallel	237 (98.8%)	79.0 (94.0%)		99.0 (99.0%)	39.0 (97.5%)		
**Post-NAC margin**			0.466			0.969	0.643
Non-Smooth	222 (92.5%)	74.0 (88.1%)		89.0 (89.0%)	35.0 (87.5%)		
Smooth	18.0 (7.5%)	10.0 (11.9%)		11.0 (11.0%)	5.00 (12.5%)		
**Post-NAC internal echo**			0.563			0.653	0.923
Homogeneous	27.0 (11.3%)	6.00 (7.1%)		13.0 (13.0%)	3.00 (7.5%)		
Non-Homogeneous	213 (88.8%)	78.0 (92.9%)		87.0 (87.0%)	37.0 (92.5%)		
**Post-NAC posterior echo**			0.0089			0.00312	0.561
Iso-Echoic	53.0 (22.1%)	33.0 (39.3%)		23.0 (23.0%)	21.0 (52.5%)		
Weaken-Echoic	187 (77.9%)	51.0 (60.7%)		77.0 (77.0%)	19.0 (47.5%)		
**Post-NAC calcification**			0.907			0.995	0.794
Coarse calcification	26.0 (10.8%)	7.00 (8.3%)		7.00 (7.0%)	2.00 (5.0%)		
Fine calcification	194 (80.8%)	72.0 (85.7%)		85.0 (85.0%)	35.0 (87.5%)		
No calcification	20.0 (8.3%)	5.00 (6.0%)		8.00 (8.0%)	3.00 (7.5%)		
**Post-NAC surrounding**			0.375			0.576	0.908

**structure distortion**							
Not distorted	213 (88.8%)	79.0 (94.0%)		93.0 (93.0%)	35.0 (87.5%)		
Distorted	27.0 (11.3%)	5.00 (6.0%)		7.00 (7.0%)	5.00 (12.5%)		
**Post-NAC blood flow type**			0.486			0.701	0.684
Central type	54.0 (22.5%)	11.0 (13.1%)		15.0 (15.0%)	9.00 (22.5%)		
No	17.0 (7.1%)+	7.00 (8.3%)		10.0 (10.0%)	6.00 (15.0%)		
Peripheral type	169 (70.4%)	66.0 (78.6%)		75.0 (75.0%)	25.0 (62.5%)		
**Post-NAC breast background**			0.891			0.812	0.923
Fatty	113 (47.1%)	37.0 (44.0%)		46.0 (46.0%)	16.0 (40.0%)		
Fibrous	127 (52.9%)	47.0 (56.0%)		54.0 (54.0%)	24.0 (60.0%)		
**Delta-length, Mean (SD), cm**	1.29 (1.52)	1.42 (1.38)	0.77	0.964 (1.24)	1.28 (1.06)	0.376	0.170
**Percentage of ultrasound length**			0.00649			0.0108	0.687
<30%	134 (55.8%)	30.0 (35.7%)		63.0 (63.0%)	14.0 (35.0%)		
≥30%	106 (44.2%)	54.0 (64.3%)		37.0 (37.0%)	26.0 (65.0%)		
**Delta-height, Mean (SD), cm**	0.839 (0.924)	1.14 (0.954)	0.0427	0.728 (0.754)	1.21 (0.768)	0.00445	0.848
**Percentage of ultrasound height**			<0.001			<0.001	0.952
<30%	108 (45.0%)	15.0 (17.9%)		47.0 (47.0%)	4.00 (10.0%)		
≥30%	132 (55.0%)	69.0 (82.1%)		53.0 (53.0%)	36.0 (90.0%)		

Note: SD, standard deviation; NAC, neoadjuvant chemotherapy; pCR, pathological complete response.

The chi-square test or Fisher's exact test was used for the nominal variable, and the Mann–Whitney test was used for the continuous variable with abnormal distribution. A two-tailed p-value <0.05 indicated statistical significance.

Supplementary Table S2. Multivariable logistic regression analysis of clinicopathological features affecting pCR.

	Training cohort			Validation cohort		
	OR-value	95%CI	P-value	OR-value	95%CI	P-value
(Intercept)	0.405	0.240-0.684	0.001	0.232	0.090-0.596	0.002
Histological grading	0.569	0.314-1.032	0.064	1.021	0.394-2.646	0.966
ER status	0.403	0.192-0.847	0.016	0.132	0.030-0.586	0.008
PR status	0.678	0.319-1.440	0.312	2.227	0.487-10.179	0.302
Her2 status	7.177	3.967-12.985	0.000	13.13	5.134-33.555	0.000

Note: ER, estrogen receptor; PR, progesterone receptor; Her2, human epidermal growth factor receptor 2; NAC, neoadjuvant chemotherapy; pCR, pathological complete response; OR, odds ratio; 95%CI, 95% confidence interval.

Supplementary Table S3. Multivariable logistic regression analysis of ultrasound features affecting pCR.

	Training cohort			Validation cohort		
	OR-value	95%CI	P-value	OR-value	95%CI	P-value
(Intercept)	0.22	0.112-0.433	0	0.131	0.039-0.437	0.001
Post-NAC posterior echo	0.437	0.25-0.763	0.004	0.269	0.113-0.637	0.003
Percentage of ultrasound length	1.551	0.873-2.756	0.134	2.259	0.966-5.283	0.06
Delta-height	0.937	0.681-1.289	0.688	1.169	0.624-2.192	0.626
Percentage of ultrasound height	3.47	1.68-7.165	0.001	6.328	1.689-23.707	0.006

Note: NAC, neoadjuvant chemotherapy; pCR, pathological complete response; OR, odds ratio; 95%CI, 95% confidence interval.
